# Mixed germ cell tumor metastatic to the skin: Case report and literature review

**DOI:** 10.1186/1477-7819-8-21

**Published:** 2010-03-23

**Authors:** Kun-Lung Chuang, Chaung-Chi Liaw, Shir Hwa Ueng, Shuen-Kuei Liao, See-Tong Pang, Ying-Hsu Chang, Heng-Chang Chuang, Cheng-Keng Chuang

**Affiliations:** 1 Division of Uro-oncology, Department of Surgery, Chang Gung Memorial Hospital, Taoyuan, Taiwan; 2 Graduate Institute of Clinical Medical Sciences, Chang Gung University, Taoyuan, Taiwan; 3 Division of Medical Oncology, Chang Gung Memorial Hospital, Taoyuan, Taiwan; 4 Department of Pathology, Chang Gung Memorial Hospital, Taoyuan, Taiwan

## Abstract

**Background:**

Testicular cancer is the most common cancer for males aged 15~35 years old. The initial presentation is typically an asymptomatic enlarged testicle. The retroperitoneum is the most common metastatic area. Other metastatic sites include the lung, liver, brain, adrenal glands, gastrointestinal tract and spleen. Skin metastasis is a rare event and frequently associated with poor prognosis.

**Case presentation:**

A 19-year old male was diagnosed testicular mixed germ cell tumor with initial presentation of cutaneous metastasis at scalp and upper abdomen. After radical orchiectomy and four courses of cisplatin-based chemotherapy, the scalp and upper abdominal lesions regressed completely. The size of lung metastases remained unchanged.

**Conclusions:**

For advanced stage testicular cancer, cisplatin-based chemotherapy is still effective to achieve partial response.

## Background

Cutaneous manifestation of an internal malignancy is rare, with an incidence of 2.9-9%[1,2]. The frequencies of skin metastases in females are 69% for breast cancer, 9% for colon cancer, and 5% for melanoma. In males the frequencies of cutaneous metastases are 24% for lung cancer, 19% for colon cancer, and 13% for melanoma [3,4] Cutaneous metastases of the genitourinary tract tumors have been associated with cancers of the prostate [5], bladder [6], and kidney [7]. This report describes a case of testicular germ cell tumor with skin metastases at the initial presentation.

## Case Presentation

**Figure 1:**
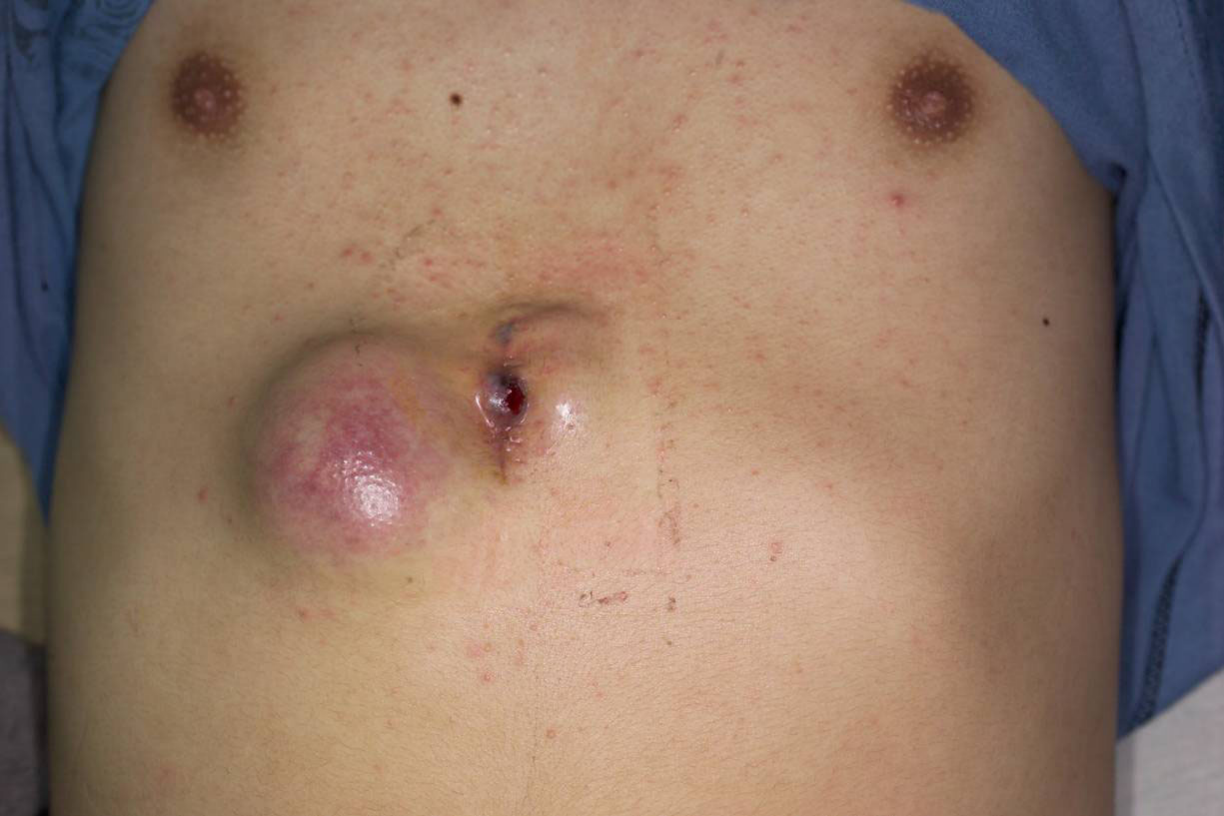
**Two upper abdominal cutaneuos nodules**.

**Figure 2:**
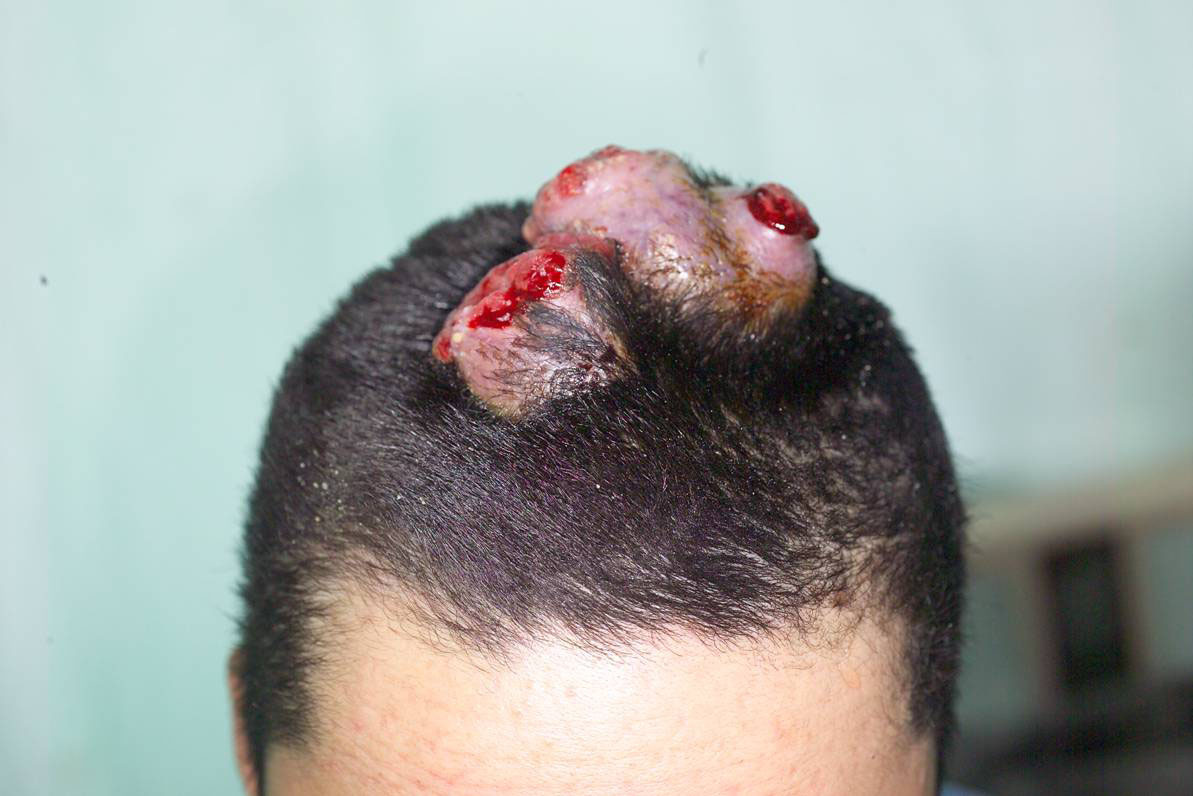
**Multiple nodules at the scalp showing ulceration and mild bleeding on the top of each nodule**.

**Figure 3:**
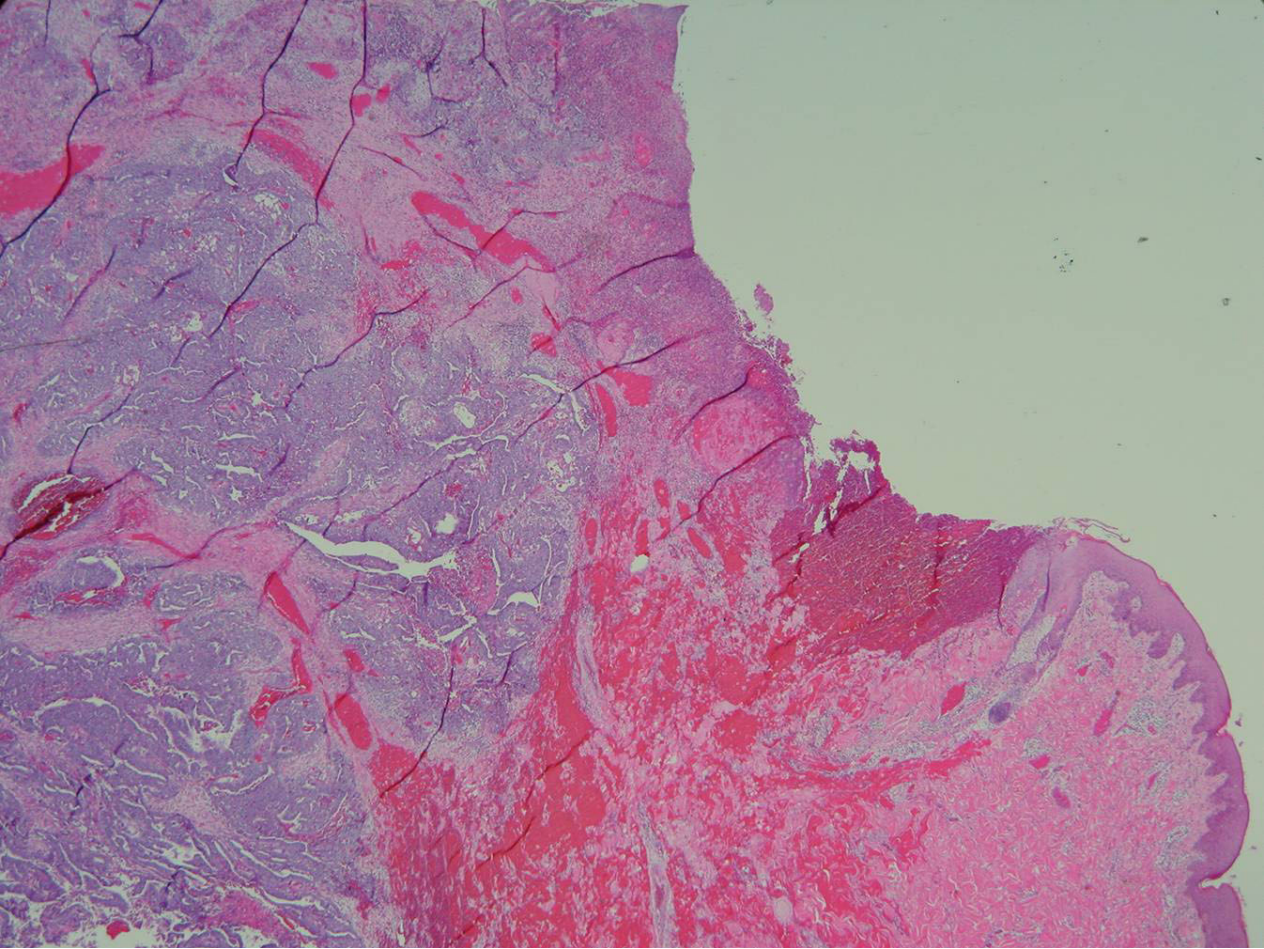
**H&E stained section of the skin revealed the ulcerated skin tumor involving the dermis and subcutis**. A tubular papillary pattern was identified at low magnification. (×20).

**Figure 4:**
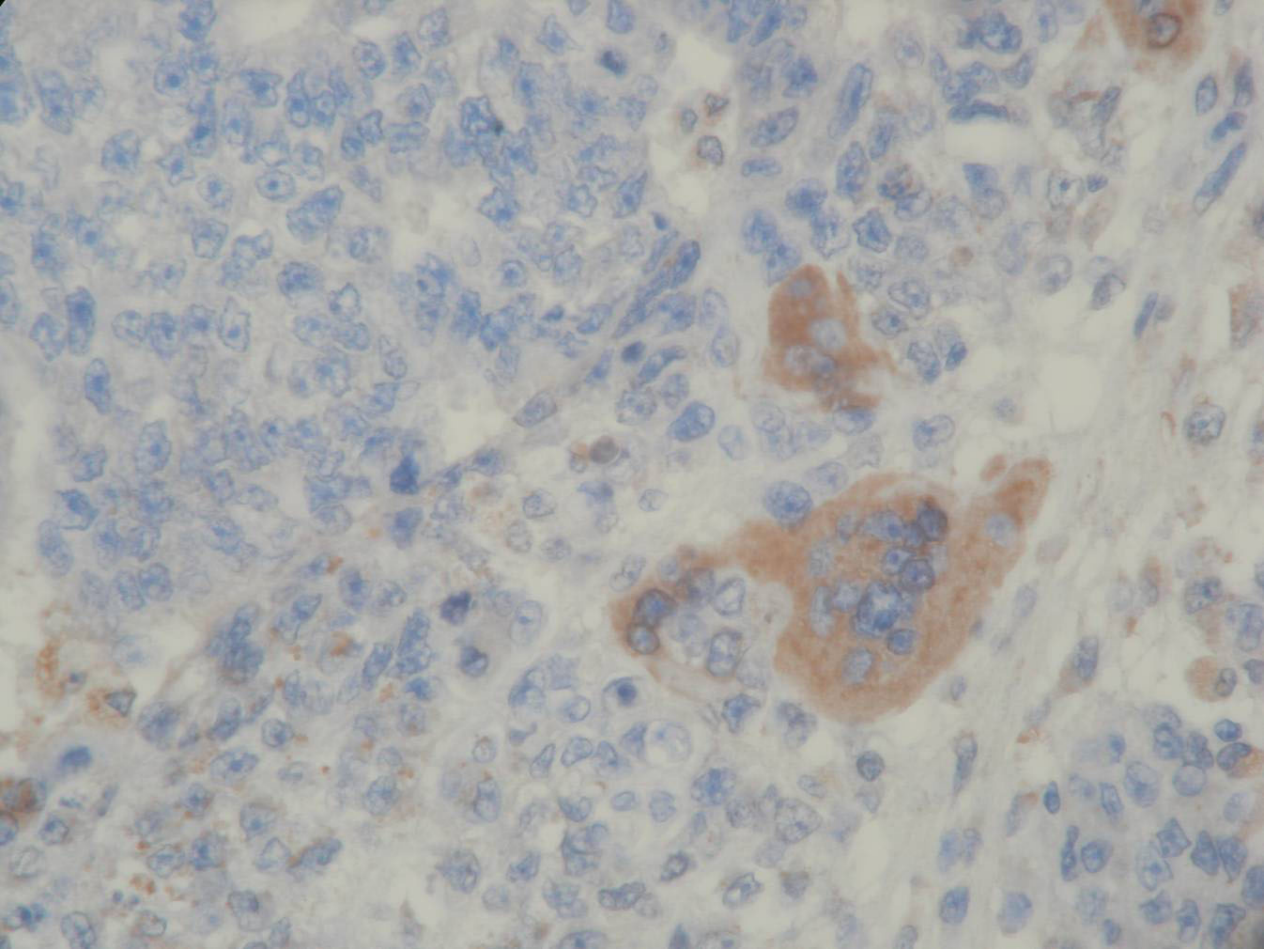
**Several syncytiotrophoblastic cells formed in the sea of other tumor cells are positive for β-HCG**. (×400).

A 19-year-old male was in good health conditions before admission. He suffered progressively enlarging upper abdominal skin lesions and scalp nodules for 3 months (fig. 1, 2). These nodules were stony hard with mild bleeding. Excisional biopsies of these two anatomical diverse origins revealed metastatic germ cell tumors. An example of H&E stained section from the abdominal lesion is shown in fig. 3. Immunohistochemical staining on the other area of the section reveals some β-HCG positive syniotrophoblastic cells in the sea of other tumor cells (fig. 4). Initial β-HCG and AFP levels in the blood were 2,630 mIU/ml and 396 ng/ml, respectively. Testicle ultrasonography disclosed two small heterogenous masses in the upper and lower poles of the left testis. By abdominal computed tomography, no obvious retroperitoneal lymph nodes were detected, but several metastatic nodules sized from 1 ~3 cm were seen in the left lower lung. Left radical orchiectomy was therefore performed. Pathology revealed that tumor cells were composed of an admixture of cystic lesions lined by squamous epithelia and containing keratin, pseudostratified columnar epithelium with goblet cells, primitive cuboidal cells, with a myxoid reticular and solid pattern, Schiller Duval bodies, hyaline globules and nests of undifferentiated epithelial cells. The primary tumor was confined in the testis. The patient underwent four courses of chemotherapy with PEB (cisplatin, etoposide, bleomycin). Complete response for the scalp lesions was achieved, while the lung metastasis remained stable four years after surgery. The two tumor markers initially detected became undetectable.

## Discussion

Cutaneous metastases originating from a solid organ are roughly 2% [1]. Most of the cutaneous metastases were noted by a prior cance diagnosis; While only 8% of cases represented the first indication of an internal malignancy [8]. The most common sites of metastastatic disease from urologic malignancies are lymph nodes, bone, lung, and liver [9]. The incidence of cutaneous metastasis from all urologic malignancies is 1.1% to 2.5% [8]. The relative incidences of metastasis to the skin among gentitourinary cancers are 3.4% to 4.0% for renal cell carcinoma, 0.84% to 3.6% for transitional cell carcinoma, 0.4% for germ cell tumors, and 0.36% to 0.7% for prostate adenocarcinoma[9].

Testicular mixed germ cell tumors are common, comprising roughly 33% of all testicular tumors. The reason for such a high incidence of testicular mixed germ-cell tumors is because germ cells in the testes are totipotent and can undergo either trophoblast or somatic differentiation. Among all the subtypes of testicular germ cell tumor, choriocarcinoma is the most aggressive with highly metastatic potential [10].

The cutaneous metastasis as the first sign of metastatic choriocarcinoma could have been either an occult or a slow growing primary testis germ cell tumor [11-15]. Cutaneous metastasis of the genitourinary malignant neoplasm is often related to advanced local extension, disseminated metastasis and poor prognosis. In this case, no retroperitoneal lymph node metastasis was observed.

According to the International Germ Cell Consensus Prognosis for Testicular Cancer[16], the 5-year progression-free survival rate is 41% for non-seminoma origin and non-pulmonary visceral metastasis. The present case was classified as poor prognostic. After four courses of PEB regimen (cisplatin, etoposide, bleomycin), the scalp and abdominal lesions achieved complete response but the lung metastasis remained stable in size.

## Conclusion

The skin is an uncommon site for testicular germ cell metastasis. A cutaneous lesion can be difficult to be differentiated from a primary cutaneous neoplasm. Excisional biopsy is required for definite diagnosis. For advanced stage testicular cancer, cisplatin-based chemotherapy is still effective to achieve biochemical remission.

## Competing interests

The authors declare that they have no competing interests with people or organizations in preparation of this study.

## Consent

Written informed consent was obtained from the patient for publication of this case report and accompanying images. A copy of the written consent is available for review by the Editor-in-Chief of this journal.

## Authors' contributions

KLC was the first author, responsible for the conception and design for the manuscript, the clinical work, the search for the literature, and the editing work.

SHU was responsible for the histopathological work. STP, HCC, YHC and CCL helped in the clinical work as well as the literature review. STP and SKL were responsible for editorship of the manuscript. CKC is the head of the department who supervised all the steps of the work.
